# Effectiveness and Safety of an Overnight Patch Containing *Allium cepa* Extract and Allantoin for Post-Dermatologic Surgery Scars

**DOI:** 10.1007/s00266-018-1172-4

**Published:** 2018-06-14

**Authors:** Welf Prager, Gerd G. Gauglitz

**Affiliations:** 1 Prager & Partner Dermatologische Praxis, Hemmingstedter Weg 168, 22609 Hamburg, Germany; 2 Department of Dermatology and Allergy, Ludwig Maximilian University, Munich, Germany

**Keywords:** Dermatologic surgery, Skin, Scar, Patch, Onion extract, Allantoin

## Abstract

**Background:**

An occlusive overnight intensive patch medical device (OIP) containing onion extract and allantoin has been developed for preventing and treating dermatologic scars and keloids. Here, we examined the efficacy and safety of the OIP for post-dermatologic surgery scars.

**Methods:**

This was an intra-individual randomized, observer-blind, controlled study in adults with post-dermatologic surgery scars. Two scars per subject were randomized to no treatment or overnight treatment with the OIP for 12–24 weeks. Scar quality was assessed using the Patient and Observer Scar Assessment Scale (POSAS) and a Global Aesthetic Improvement Scale.

**Results:**

A total of 125 subjects were included. The decrease in observer-assessed POSAS from baseline was significantly greater for treated than untreated scars at week 6 (*p* < 0.001) and 24 (*p* = 0.001). The decrease in patient-assessed POSAS was significantly greater for the treated scar than the untreated scar at week 12 (*p* = 0.017) and 24 (*p* = 0.014). Subject- and investigator-evaluated Global Aesthetic Improvement Scale scores were higher for the treated than the untreated scar at all visits. All subjects considered the global comfort of the OIP to be good or very good, and no safety concerns were identified.

**Conclusions:**

This study confirmed that the OIP safely promotes scar healing after minor dermatologic surgery.

**Level of Evidence II:**

This journal requires that authors assign a level of evidence to each article. For a full description of these Evidence-Based Medicine ratings, please refer to the Table of Contents or the online Instructions to Authors www.springer.com/00266.

## Introduction

Following surgical procedures or injury, cutaneous scars can develop due to production of collagen-rich connective tissue [1]. These scars may be accompanied by redness, itching, pain, and restricted mobility of the skin. Typically, after a few weeks, the scar matures, becoming lighter and narrower, although full maturation of a scar may take up to 2 years. In some cases, however, cutaneous scars can become hypertrophic or result in keloids.

Preventing pathological scarring and keloids is much easier that treating them later and should be started as early as possible after the injury or surgery [2–4]. Ointments containing onion extract and occlusion by silicone sheets or gels are commonly recommended to prevent excessive scarring [4, 5]. Other noninvasive measures include topical compounds, tapes, compression bandages, physiotherapeutic measures, and therapeutic ultrasound [4, 6, 7]. Invasive measures for treating scars and keloids include injections with various agents, cryotherapy, laser therapy, and other surgical procedures. Topical treatments, in particular, are considered appropriate for all kinds of scars, irrespective of scar size or patient age [7].

A topical gel containing onion (*Allium cepa*) extract and allantoin has been available for more than 60 years treating, preventing, and reducing dermatologic scars and keloids [8]. Onion extract is reported to have anti-inflammatory, anti-microbial, anti-proliferative, and regenerative activities [7], while allantoin is reported to have keratolytic, hydrating, epithelizing, and anti-irritant activities [9]. Several clinical trials have confirmed that this gel is well tolerated and helps prevent pathological scarring and improves preexisting scars [8, 10–16].

Combining onion extract and allantoin with occlusion has been reported to provide optimal results [15]. An occlusive overnight intensive patch medical device (OIP) containing onion extract and allantoin was recently developed to provide optimal scar healing and improved convenience for the patient. The OIP features an occlusive active release liner with an adhesive layer separated by a micro-air cushion seal, and it can be cut to size for small scars or placed side-by-side for larger scars. In contrast to the gel, which must be applied twice daily, the OIP only needs to be applied once daily.

Here we describe the results of an intra-individual randomized controlled study confirming the efficacy and safety of the OIP for treating post-dermatologic surgery scars.

## Methods

### Study Design and Ethics

This was an intra-individual randomized, observer-blind, controlled study to examine the effectiveness and safety of the OIP over 24 weeks in post-dermatologic surgery scars. The study was performed at nine sites in Germany between October 2013 and July 2015. The study was reviewed by the *Ethik*-*Kommission der Ärztekammer* (Hamburg, Germany), and the clinical trial investigation plan and related documents were reviewed and approved by the local ethics committees where required. The study was conducted in accordance with the International Conference on Harmonization Good Clinical Practice guidelines and the Declaration of Helsinki. All subjects provided written informed consent to take part in the study.

## Subjects

Adults (≥ 18 years of age) who had undergone dermatologic surgery were considered for inclusion in the study if they had at least two newly formed scars 1–10 cm in length on similar body regions. The subjects had to be enrolled within 3 weeks after the surgery but after the wounds were closed, the scars were controlled and, if applicable, the suture material had been removed. Included subjects could not have had a known history of keloids or hypertrophic scars. Additional exclusion criteria included dermatologic resurfacing procedures or noninvasive skin-tightening procedures, such as medium or deep chemical peeling, dermabrasion, or laser therapy in the area intended to be treated within 8 weeks before enrollment; an infection, a wound, or eczema in the skin area intended to be treated; known hypersensitivity to any ingredient in the OIP; or an ongoing severe or uncontrolled systemic disease, malignant tumor, or known human immunodeficiency virus infection. Subjects could also not be taking or planning to take systemic corticosteroids or topical corticosteroids for the skin area intended to be treated. Women could not be pregnant or breastfeeding and, if of childbearing potential, had to be using an effective method of birth control.

### Study Conduct and Assessments

At the baseline visit (day 1), the investigator selected two similar scars from comparable skin areas excluding the upper back (to reduce the risk of scar dehiscence). The scars were randomized 1:1 to the OIP (Contractubex^®^ Overnight Intensive Patch, Merz Pharmaceuticals GmbH) or no patch via a computerized randomization program (RANCODE Version 3.6, IDV Datenanalyse und Versuchsplanung, Gauting, Germany). Subjects were not blinded, but the investigators were blinded to the randomization and treatment received for each scar. For scars treated with the OIP, every night before going to bed, subjects cleaned and dried the scar and applied a new OIP covering the scar completely. The OIP had to be left in place overnight for at least 6 h and up to 12 h. Subjects had to continue treatment for at least 12 weeks, although they could choose to continue for an additional 12 weeks. During the study, subjects could not apply systemic or topical corticosteroids to the scars; undergo dermatological resurfacing or noninvasive skin-tightening procedures including medium/deep chemical peeling, dermabrasion, or laser therapy in the treatment area; apply topical agents containing active ingredients (e.g., self-tanning agents); receive excessive sun exposure (e.g., outdoor work or tanning bed); or apply hydrating lotions in the treated area.

At baseline (day 1), week 6, week 12, and (if the subject remained in the study) week 24 visits, the investigators assessed scar quality using the observer-evaluated Patient and Observer Scar Assessment Scale (POSAS), and the subjects completed the patient-evaluated POSAS. Throughout the study, investigators and all other individuals involved in conducting it were blinded to which scar had been treated with the OIP. The observer-evaluated POSAS includes vascularization, pigmentation, thickness, relief, and pliability, each scored on a scale from 1 (normal skin) to 10 (worst imaginable skin) so that the total score ranges from 5 (best scar appearance) to 50 (worst scar appearance), and the patient-evaluated POSAS includes pain, itching, color, stiffness, thickness, and irregularity, also scored from 1 to 10 (least severe to most severe) so that the total score ranges from 6 (best scar appearance) to 60 (worst scar appearance) [17].

At all post-baseline visits, subjects and investigators also completed the Global Aesthetic Improvement Scale (GAIS) [18], in which 1 = worse, 2 = no change, 3 = improved, 4 = much improved, and 5 = very much improved. In addition, subjects completed a global comfort assessment scale (1 = very good; 2 = good; 3 = moderate; 4 = poor) and rated their satisfaction with scar appearance (satisfied, partially satisfied, or dissatisfied).

Adverse events (AEs) were recorded by investigators at each visit along with severity, relationship to the treatment, outcome, and expectedness.

### Study Size

The sample size was estimated based on the results of a previous investigation with Contractubex^®^ gel (Merz Pharmaceuticals, unpublished observations). With a mean change from baseline in POSAS of − 8.7 ± 5.65 for the gel and − 6.1 ± 6.71 for untreated scars and a correlation of − 0.2, 120 randomized subjects were estimated to provide 83.7% power to show a significant difference at *α* = 0.05 using a two-sided paired *t* test.

### Statistical analysis

The primary effectiveness variable was the change in the observer-evaluated POSAS between baseline and week 12. Secondary effectiveness variables included the absolute change from baseline in the total score of the observer-evaluated POSAS to weeks 6 and 24; the absolute change from baseline in the total score of the patient-evaluated POSAS to weeks 6, 12, and 24; and the investigator- and subject-evaluated GAIS at weeks 6, 12, and 24. Differences in POSAS scores between OIP-treated and untreated scars were examined by analysis of covariance, with treatment and pooled site as fixed effects, subject as a random effect, and missing data replaced using the last observation carried forward. The main analyses were performed in the full analysis set, which was defined as all subjects for whom the primary effectiveness variable was available. In a post hoc analysis, differences in GAIS between groups were analyzed by analysis of variance, with treatment and pooled site as fixed effect and subject as random effect.

The primary safety assessment was the occurrence of treatment-emergent AEs (TEAEs), which was calculated for all subjects exposed to the OIP at least once.

All analyses were performed using SAS version 9.2 or higher (SAS Institute, Cary, NC, USA). A two-sided p-value below 0.05 was considered to indicate statistical significance.

## Results

### Subjects

**Fig. 1:**
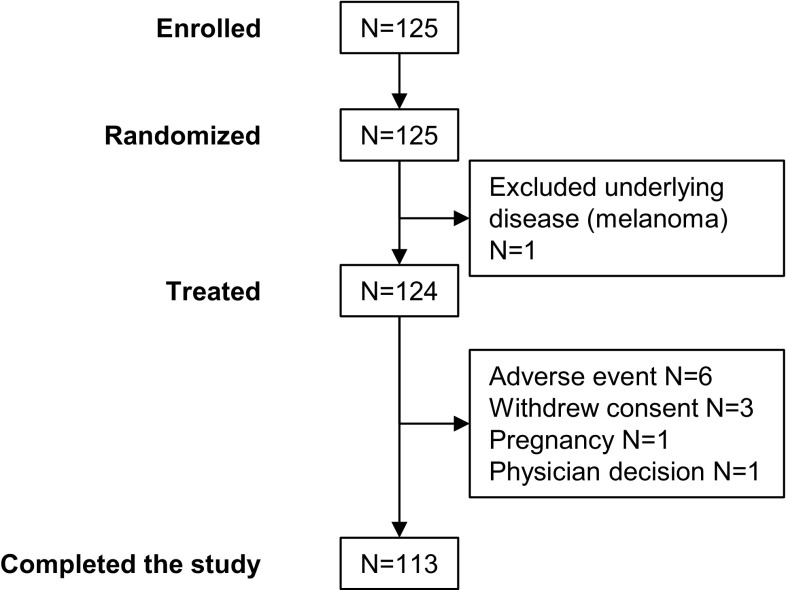
Disposition of subjects in the study. A total 125 adults who had undergone dermatologic surgery were included in the study. Two newly formed scars on each subject were randomized to treatment with the OIP or no treatment for at least 12 weeks, although subjects could choose to continue for 24 weeks

A total of 125 subjects were included between October 2, 2013 and February 12, 2015, and the study was completed on July 30, 2015. One enrolled subject was not treated because of an excluded medical condition (melanoma). Of the 124 treated subjects, 113 completed the study (Fig. 1). All subjects were Caucasian, and just over half were men (*n* = 68; 54.8%). Ages ranged from 20 to 86 years (mean ± standard deviation, 48.6 ± 15.7). Body mass indices ranged from 17.3 to 36.0 (mean ± standard deviation, 25.0 ± 3.6).

### Treatment Effectiveness

**Fig. 2:**
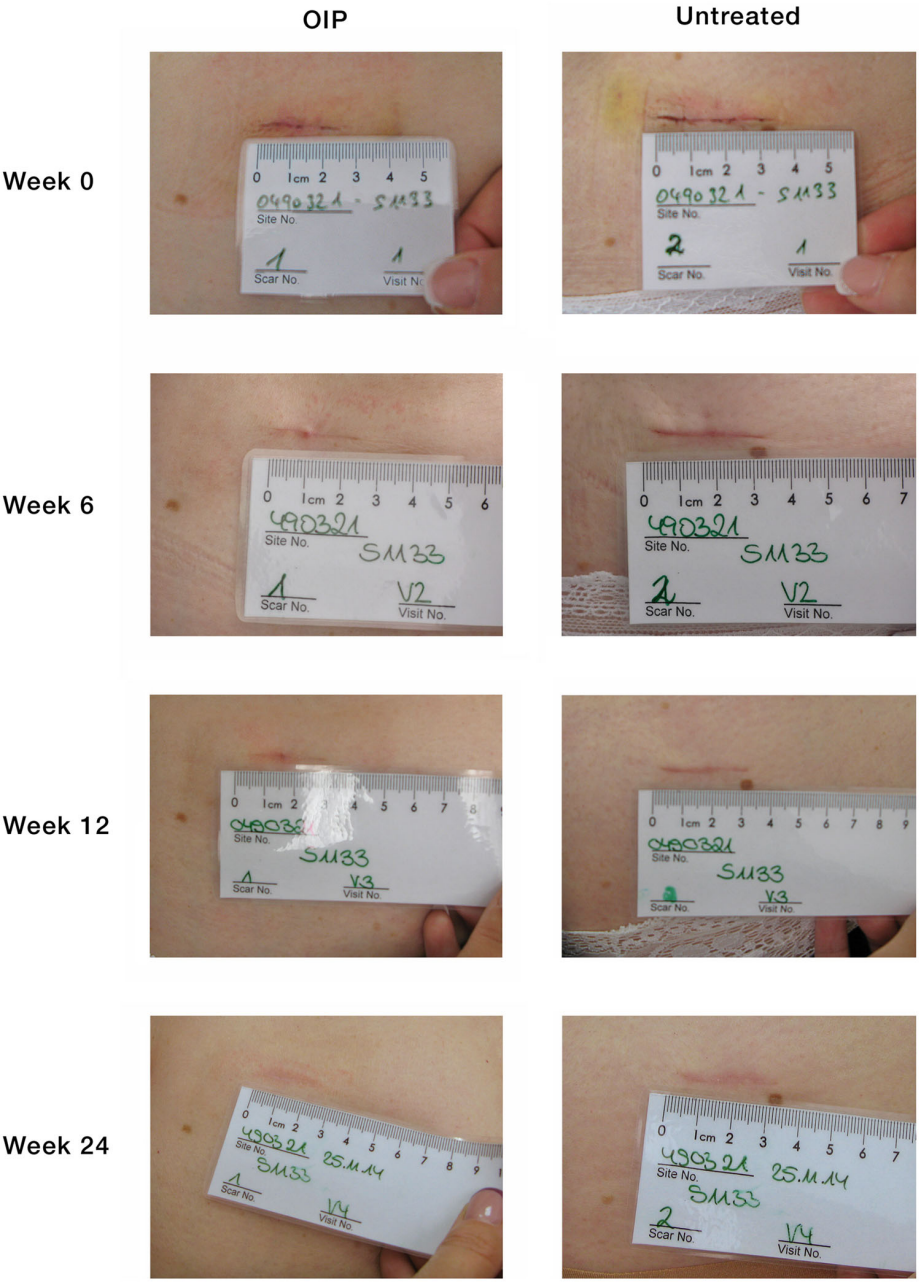
Photographic images from a single subject’s scars at baseline (week 0) and weeks 6, 12, and 24. Abbreviation: OIP, overnight intensive patch

Scars were assessed at baseline (day 1) and weeks 6, 12, and 24 by blinded observers (based on digital photographs) and by the subjects. A typical series of digital images from a single subject is shown in Fig. 2.

#### POSAS

**Fig. 3:**
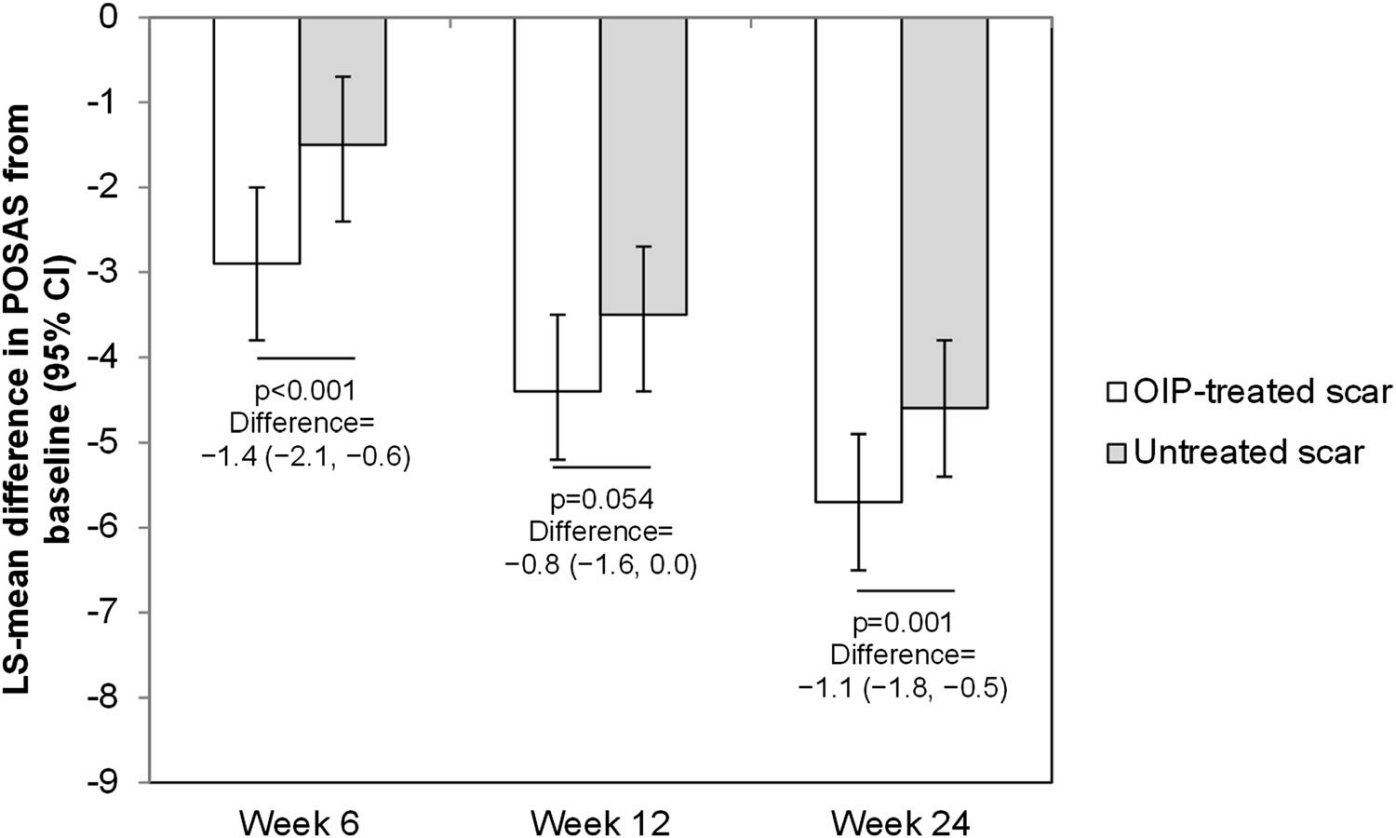
Changes from baseline in observer-evaluated POSAS. At baseline and weeks 6, 12, and 24, blinded observers completed the observer-assessed POSAS. Values are for the full analysis set (*N* = 124). *P* values were determined by analysis of covariance with treatment and pooled site as fixed effect and subject as random effect and with the value at baseline as the covariate. Abbreviations: *CI* confidence interval, *LS* least square, *OIP* overnight intensive patch, *POSAS* Patient and Observer Scar Assessment scale

At week 12, the blinded observer-evaluated POSAS, the primary outcome measure, decreased for both the treated and the untreated scars (Fig. 3). The decrease from baseline was not significantly different between treated and untreated scars at week 12 (*p* = 0.054), although it was significantly greater for treated scars than untreated scars at week 6 (*p* < 0.001) and week 24 (*p* = 0.001).

**Fig. 4:**
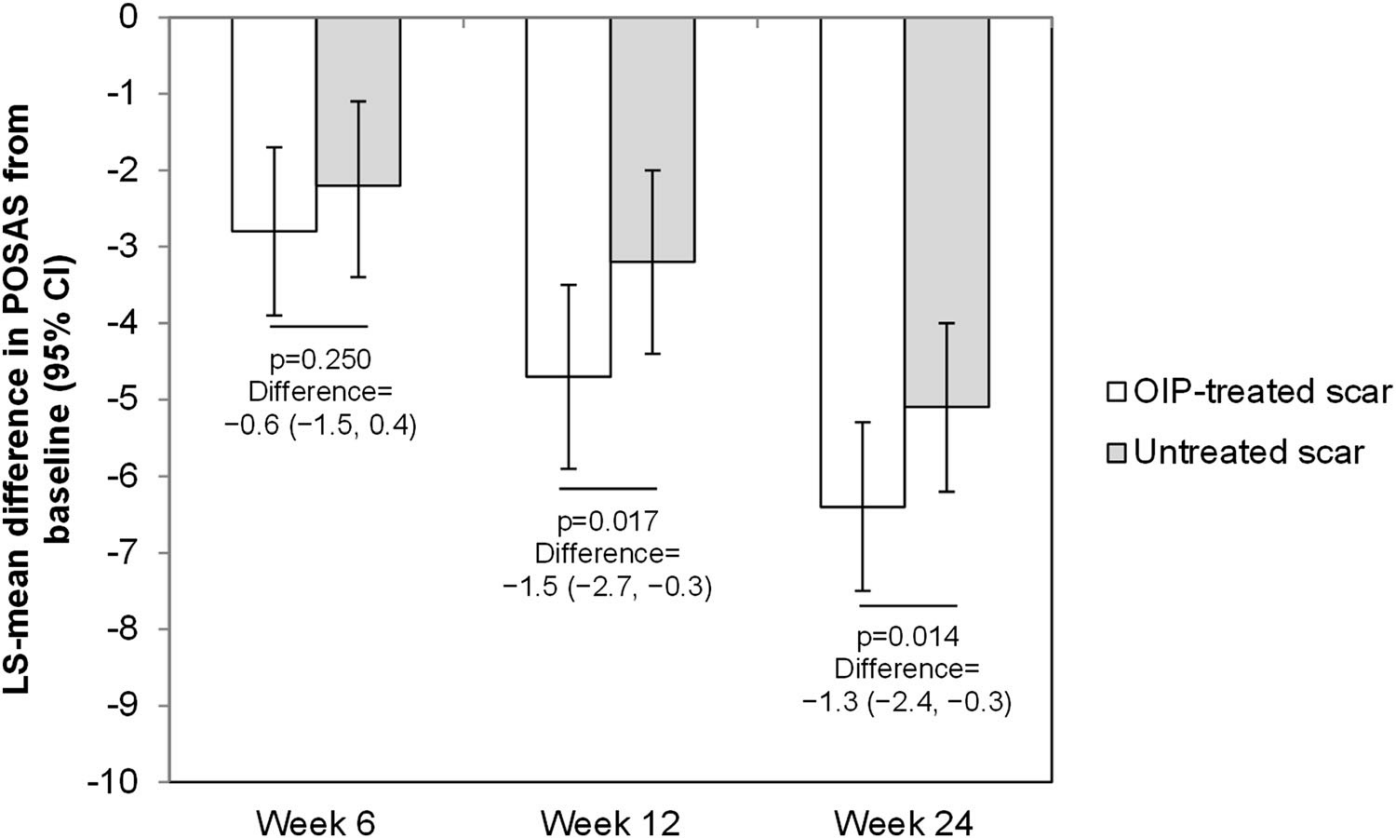
Changes from baseline in patient-evaluated POSAS. At baseline and weeks 6, 12, and 24, study subjects completed the patient-assessed POSAS. Values are for the full analysis set (*N* = 124). *P* values were determined by analysis of covariance with treatment and pooled site as fixed effect and subject as random effect and with the value at baseline as the covariate. Abbreviations: *CI* confidence interval, *LS* least square, *OIP* overnight intensive patch, *POSAS* Patient and Observer Scar Assessment scale

For patient-evaluated POSAS, the decrease from baseline was significantly greater for treated scars than for untreated scars at week 12 (*p* = 0.017) and week 24 (*p* = 0.014), but not at week 6 (*p* = 0.25) (Fig. 4).

#### GAIS

The subject- and investigator-evaluated GAIS increased from baseline for both treated and untreated scars (Table 1). A post hoc analysis revealed that the increase from baseline was significantly greater for the treated scar than for the untreated scar at all visits for both investigators (*p* = 0.011 at week 6, 0.001 at week 12, and 0.044 at week 24) and subjects (*p* < 0.001 at weeks 6, 12, and 24).

**Table 1:** Investigator- and subject-evaluated GAIS

Evaluator	Week	Least square mean GAIS score (95% CI)		Least square mean difference (95% CI)	*p* value treated versus untreated
		OIP-treated scar	Untreated scar		
Investigator	6	3.2 (3.1, 3.4)	3.0 (2.8, 3.2)	0.2 (0.1, 0.4)	0.011
	12	3.5 (3.3, 3.7)	3.2 (3.0, 3.4)	0.3 (0.1, 0.5)	0.001
	24	3.9 (3.7, 4.2)	3.6 (3.4, 3.8)	0.4 (0.2, 0.5)	< 0.001
Subject	6	3.3 (3.1, 3.4)	2.9 (2.7, 3.0)	0.4 (0.2, 0.6)	< 0.001
	12	3.6 (3.4, 3.8)	3.0 (2.8, 3.2)	0.5 (0.3, 0.7)	< 0.001
	24	3.8 (3.6, 4.1)	3.4 (3.1, 3.6)	0.5 (0.3, 0.7)	< 0.001

At weeks 6, 12, and 24, blinded investigators and subjects rated improvement in scars using the GAIS (1 = worse, 2 = no change, 3 = improved, 4 = much improved, 5 = very much improved). *p* values were determined in a post hoc analysis by analysis of variance with treatment and pooled site as fixed effect and subject as random effect. Values are for the full analysis set (*N* = 124). Abbreviations: *CI* confidence interval, *GAIS* Global Aesthetic Improvement Scale, *OIP* overnight intensive patch

### Satisfaction and Comfort

Subjects were more often satisfied with the healing of their treated scars than with the healing of their untreated scars (80.7 vs. 68.1% at week 6; 85.7 vs. 72.3% at week 12; 78.2 vs. 66.4% at week 24), and most subjects reported their global comfort to be good or very good for the treated scar (94.1% at week 6, 94.2% at week 12, 86.6% at week 24) (data not shown).

### Safety

Of the 124 treated subjects, 22 reported TEAEs that were assessed by investigators as related or possibly related to the OIP. The most common were erythema (*n* = 11) and pruritus (*n* = 12) at the application site. Other TEAEs considered to be possibly or probably related to the OIP included application-site eczema (*n* = 1), blister (*n* = 1), dermatitis (*n* = 1), contact dermatitis (*n* = 1), skin irritation (*n* = 1), swollen face (*n* = 1), and wound dehiscence (*n* = 1). All of these events were mild or moderate in severity.

Six subjects (4.8%) had AEs leading to discontinuation, four of which were considered treatment-related. These treatment-related AEs included one subject who had dermatitis around the treated scar that worsened from mild to moderate within 4 days but resolved after discontinuing treatment; one subject with a history of pollinosis who had a swollen face and pruritus outside of the application site that started after 1 week and worsened from mild to moderate 3 weeks later; one subject with a history of allergy of unspecified type who had mild erythema and pruritus at the application site after 6 weeks of treatment that continued to worsen after stopping treatment; and one subject with moderate eczema at the application site after 2 weeks that led to withdrawal of consent. No treatment-related serious adverse events were reported, and no other safety concerns were identified.

## Discussion

This study examined an OIP that combines the benefits of occlusion and onion extract and allantoin. The study confirmed that the OIP significantly improves the healing of post-dermatologic scars. The study also confirmed that the OIP was safe, well tolerated, and well accepted.

Confirmation of efficacy was based on a robust primary outcome measure, the observer-blinded POSAS, a validated score for assessing scars [17]. Differences between the treated and control scars were statistically significant at weeks 6 and 24. This was further supported by comparisons of patient evaluations using the POSAS [19] as well as by investigator and patient evaluations using the GAIS [20, 21]. In addition, the study was designed to minimize bias and confounders and to ensure reliable and relevant effectiveness and safety results. In particular, the study design included an intra-individual randomized controlled comparison to ensure that each pair of scars was as comparable as possible. In addition, a validated [19], observer-blinded assessment was used as the main endpoint. Subject assessments were open to bias by expectations, although they mirrored the results of the blinded observer assessments. The semi-blinded design could have resulted in some accidental unblinding, although no cases were reported.

Our results agree with other studies showing that the onion extract and allantoin used in the OIP are effective and well tolerated for treating, reducing, and preventing hypertrophic scars and keloids [8, 10–16]. Mild-to-moderate irritation at or eczema around the treated scar were reported by a few patients, most of whom had allergies. Patients with allergies should therefore be monitored for possible reactions to the OIP.

This study had some limitations. First, this study did not compare the effects of the OIP with occlusion alone or with gel alone, so it did not allow conclusions to be drawn about relative efficacy or safety or about the contributions of each component to overall efficacy. Rather, the study was intended to confirm that the OIP improved scar healing and that it is well accepted and tolerated by patients. A previous study by Hosnuter et al. [15], however, found that optimal results are obtained using the combination of topical onion extract–allantoin gel and a silicone gel sheet. A second limitation is that within-treatment comparisons between post-treatment and baseline measurements could have been biased by the observer’s expectations, especially for subjects, who knew which scar had been treated with the OIP. These biases, at least for the investigator's assessments, should have been accounted for by blinding and randomization. A further potential limitation is that the study did not meet the primary objective of showing a significant difference in the observer-blinded POSAS at week 12. However, significant differences were detected at weeks 6 and 24, and according to investigators and subjects, the treated scar was more improved than the untreated scar at all time points. This lack of a significant difference at week 12 in the observer-blinded POSAS could be due to the dynamics of scar healing combined with the limitations of digital imaging.

In conclusion, the current study confirmed that the OIP can be safely used to promote scar healing after minor dermatologic surgery. The device is convenient and comfortable, making it an excellent choice for dermatologists and their patients.
